# The Impact of Social Participation Interventions on Quality of Life of Older Adults in Residential Aged Care Facilities: A Systematic Review

**DOI:** 10.1111/jocn.70483

**Published:** 2026-08-04

**Authors:** Sara Habibian, Guogui Huang, Nasir Wabe, Johanna I. Westbrook, Amy D. Nguyen

**Affiliations:** ^1^ Centre for Health Systems and Safety Research, Australian Institute of Health Innovation Macquarie University Sydney New South Wales Australia; ^2^ St Vincent's Clinical Campus UNSW Sydney Kensington New South Wales Australia

**Keywords:** older adults, quality of life, residential aged care home, social participation

## Abstract

**Aim:**

To examine the impact of social participation interventions on the quality of life of older adults in residential aged care home settings.

**Design:**

Systematic review.

**Methods:**

The inclusion criteria were articles published in English language between 2015 and 2025, peer‐reviewed articles, availability of full‐text, and conducted in residential aged care home settings, aged 65 years and over. Quality appraisal of articles was conducted using the Mixed Methods Appraisal Tool.

**Data Sources:**

A systematic search was conducted in MEDLINE, Embase, Scopus, PsycINFO, and CINAHL.

**Results:**

Ten studies met the inclusion criteria. Four studies showed correlations between quality of life scores and social participation engagement during activities involving virtual reality tourism, resistance training, Sinaki exercises, and combined person‐centred care with social interaction. Improvements in quality of life were associated with six interventions including virtual reality tourism, resistance training, Sinaki exercises, tai chi exercise, and two distinct multicomponent person‐centred care programmes involving pain management, communication, organisation of activities and medication review. Quality appraisal showed studies were of moderate to high quality.

**Conclusions:**

The review identified several social participation interventions that were associated with enhanced quality of life for older adults in residential aged care homes. Adoption of these interventions, including a multi‐component approach, is recommended to optimise resident outcomes.

**Patient or Public Contribution:**

No Patient or Public Contribution was involved in this study.

**Impact:**

Evidence on the effectiveness of social participation interventions on quality of life in aged care homes remains limited and fragmented. This review identified six key interventions that improve aged care residents' quality of life, offering valuable insights for policies and practices aimed at enhancing the quality of life of residents in residential aged care homes.

**Reporting Method:**

This systematic review followed the PRISMA 2020 guidelines and was reported according to the SWiM (Synthesis Without Meta‐analysis) reporting guideline recommended by the EQUATOR Network.

**Trial Registration:**

https://www.crd.york.ac.uk/PROSPERO/myprospero; identifier: CRD420250646145

## Abstract (highlights)

**Highlights:**

- Social participation interventions can improve quality of life in aged care residents.
- Quality of life benefits were identified across a range of social participation intervention types.
- Indoor and non‐exercise activities were the most frequently studied social participation interventions.

**What Does This Paper Contribute to the Wider Global Clinical Community?:**

- This study identified several social participation interventions that significantly influenced the quality of life in residential aged care homes.
- This study provides evidence‐based guidance for global aged care practice and policy.

## 1 Introduction

Population ageing is a significant global phenomenon, particularly prevalent in developed nations. According to the World Health Organisation (WHO), the global population aged 60 years and over will increase from 1 billion in 2020 to 1.4 billion in 2030, and will more than double to 2.1 billion by 2050 (WHO 2024). As a result of declining health and cognitive abilities in the ageing process, the focus is shifting from merely extending lifespan to enhancing the quality of life (QOL) in later years—a goal increasingly shared by both older adults and aged care providers (Hutchinson et al. 2024; Irlam 2018). In response, the philosophy and practice of person‐centred care are being widely adopted across the aged care sector (Oppert et al. 2018).

The WHO defines QOL as an individual's perception of their position in life within the context of their culture, value systems, goals, expectations, standards, and concerns (Teoli 2023). QOL broadly reflects wellbeing, happiness, and life satisfaction (Advisers ACF 2024). It is a subjective and multifaceted concept, encompassing several domains like physical health, psychological wellbeing, functional independence, social relationships, environmental context, and personal beliefs (Conde‐Sala et al. 2017). These domains are dynamic and constantly interact with one another, with changes in one domain affecting others, ultimately shaping an individual's overall QOL (van Leeuwen et al. 2019).

Social participation is widely recognised as a key determinant of QOL in later life (Döhring et al. 2024). It is commonly defined as a person's involvement in activities that provide interaction with others in the community based on a person's time, resources, societal context, and personal values (Levasseur et al. 2022). This includes a broad range of activities, including social, recreational, cultural, civic, or religious engagement, as well as maintaining meaningful interpersonal relationships (Levasseur et al. 2010). Social participation significantly influences multiple QOL domains by fostering a sense of purpose and belonging and is consistently associated with higher life satisfaction (Döhring et al. 2024). Interventions that promote social participation have been shown to support healthy ageing by improving cognitive function, reducing depressive symptoms, enhancing self‐rated health and physical functioning, and even lowering the risk of falls among older adults (Douglas et al. 2017; Wei et al. 2024).

Despite these benefits, participating in social activities can be difficult for older adults due to physical and mental decline during ageing. Older people living in residential aged care homes (RACHs) are particularly vulnerable to a decline in social engagement (Sedlackova et al. 2024) as their multiple chronic health issues mean that they can require support for daily activities (Quan et al. 2020). The transition from home to institutional care often disrupts established social networks, leading to a loss of connection with long‐standing friends and neighbours. While RACHs may offer structured opportunities for social engagement, residents may hesitate to participate because of fear of unfamiliar settings, language or cultural barriers, and experiencing grief over the loss of friends within the facility (Hutchinson et al. 2024; Akhtar 2024). This reluctance can lead to social isolation, which is not only associated with reduced QOL (Miki et al. 2024), but also increases the risk of cognitive decline (Holt‐Lunstad et al. 2015). Notably, the prevalence of profound isolation among RACH residents is estimated to be at least twice as high as that of older adults living in the community (Victor 2012).

Social participation interventions refer to structured activities or programmes specifically designed to enhance individuals' engagement in meaningful social interactions and community life. These interventions may include one‐to‐one personal contact or group‐based activities such as art, music, topical discussions, or exercise (O'Rourke et al. 2018). Effective social participation interventions typically include both social contacts with valued others—such as friends, family, and staff—and participation in shared social activities (Newlin et al. 2015), with a primary purpose to reduce social isolation, improve mental health and wellbeing, and foster a sense of belonging and inclusion (Scope 2023).

Numerous studies have explored the effectiveness of social participation interventions in RACHs, reporting potential benefits for residents' QOL. Examples include humour therapy (Brodaty et al. 2014), communication technology tools (Akhtar 2024), student placements programmes (Annear et al. 2017), and social robotics (Chen et al. 2020). However, the existing evidence on whether and which social participation interventions are effective remains limited and fragmented, with gaps regarding which interventions are most beneficial and under what conditions. A more comprehensive understanding of the effectiveness of social participation interventions, the underlying mechanisms, contextual factors, and resident preferences that influence participation is critical for tailoring effective strategies.

This systematic review hence aims to synthesise and critically appraise existing evidence regarding the impact of social participation interventions on QOL among older adults living in RACHs. The findings will provide essential evidence regarding the effectiveness of different types of social participation interventions, crucial in informing policies and practices to enhance the QOL of RACH residents.

## 2 Methods

### 2.1 Protocol

This systematic review was registered in PROSPERO, followed the PRISMA 2020 guidelines (Page et al. 2021) and was reported according to the SWiM (Synthesis Without Meta‐analysis) reporting guideline from the EQUATOR network (Campbell et al. 2020). The research question was formed according to PICO (population, intervention, comparison and outcome) (Schardt et al. 2007).

### 2.2 Search Strategy

Information on social participation strategies to improve QOL for residents in RACHs was collated from five electronic databases: MEDLINE, Embase, Scopus, PsycINFO, and CINAHL. Reference lists of included studies were screened for potentially eligible articles. The search strategy consisted of concepts about ‘residential aged care home’, ‘social participation’ and ‘QOL’ (Table S1) and was developed after consulting with a specialised academic librarian in health and medicine. Additionally, the full search strategy for MEDLINE (Ovid) is provided in Table S2 as exported directly from the database search history.

### 2.3 Inclusion and Exclusion Criteria

Peer‐reviewed articles written in English, published between Jan 2015 and Jan 2025, were included. Eligible articles focused on older adults (aged ≥ 65 years) residing in RACHs, with a social participation intervention and reported on QOL. Interventional comparative studies (e.g., before and after, pre‐ and post‐, RCTs) were included, while qualitative studies, systematic reviews and meta‐analyses were excluded. According to the International Classification of Functioning, Disability and Health (ICF) in 2001 (Organization WH 2007), we considered social participation as involvement in life situations. All studies were selected based on the ICF framework, focusing on interventions that actively provided opportunities for residents to engage socially or encouraged them to initiate meaningful interactions.

We did not apply strict intervention‐type criteria due to the substantial heterogeneity of social participation interventions. We aimed to capture a broad range of interventions, according to the definition of social participation, to better understand their effectiveness on the quality of life of aged care residents.

### 2.4 Screening

All identified citations were exported in EndNote, in which duplicates were removed and the remaining were transferred to Rayyan (Ouzzani et al. 2016), a web‐based platform for reviewing articles. Two independent reviewers (SH, AN) screened all titles and abstracts according to inclusion and exclusion criteria, and any conflicts during screening were resolved through discussion or, if required, by a third reviewer (NW). Full texts of included articles were then screened by the same two independent reviewers (SH, AN). Any disagreements regarding the studies' outcomes, design, and population were resolved through consensus, or if disagreement persisted, by the decision of a third reviewer (NW).

### 2.5 Data Extraction

After full‐text review, the primary reviewer (SH) extracted data from all included studies. A standardised data extraction template including study characteristics (e.g., author, year, country), study design, population details (e.g., age, sample size), interventions, QOL assessment tools, outcome measures, and main findings in an Excel spreadsheet format was used to collect relevant data consistently from each included study. The research team reviewed the extracted data to ensure accuracy and consistency and to confirm that the process was proceeding as expected.

### 2.6 Quality Appraisal

A rigorous critical appraisal of the included studies was conducted to evaluate the quality of the evidence, using the Mixed Methods Appraisal Tool (MMAT) version 2018, a widely used quality appraisal tool for systematic reviews (Hong, Gonzalez‐Reyes, and Pluye 2018). The overall inter‐rater reliability of the MMAT ranged from moderate to perfect agreement (Pace et al. 2012). As this review included multiple study designs including randomised controlled trial, cluster randomised controlled trial, quasi‐experimental designs, and mixed method study, MMAT was selected since it is designed to assess all types of studies and it can provide a more efficient appraisal with consistent criteria (Hong, Fàbregues, et al. 2018). The results are reported descriptively across the five domains of MMAT (Hong et al. 2020). SH completed the MMAT checklist for each article. Results were then reviewed and confirmed by another researcher (GH). Any conflicts were resolved through discussion with another reviewer (AN).

### 2.7 Synthesis

A synthesis without meta‐analysis was conducted due to the anticipated heterogeneity in study designs, QOL measurement tools, and social participation interventions. Results were synthesised narratively by assessing the effects of interventions (positive, negative, or mixed), the consistency of results across similar intervention types, and the timing of effects during follow‐up. According to the SWiM reporting, this approach enables meaningful interpretation despite observed heterogeneity. Findings were systematically organised based on QOL domains (e.g., emotional wellbeing, social connection, and activities) and the types of social participation interventions examined. Three subgroups were examined including type of social participation, resident characteristics, and intervention factors.

## 3 Results

### 3.1 Study Selection

The PRISMA flowchart represents the process and the number of studies included in this systematic review (Figure 1). The result of our database search revealed a diverse range of 1285 articles. After identifying and removing 391 articles as duplicates, there were 894 articles remaining for title and abstract screening. Then, 857 papers were excluded as not meeting the inclusion criteria during title and abstract screening. In the final stage of screening, 37 articles were reviewed in full text, and 10 articles were considered eligible for data extraction. The main reasons for excluding articles were: not reporting the impact of social participation interventions on QOL (*n* = 11), non‐RACH setting (*n* = 8), non‐intervention study (i.e., absence of pre‐post intervention test) (*n* = 6), and unrelated outcome‐ (*n* = 2).

**FIGURE 1:**
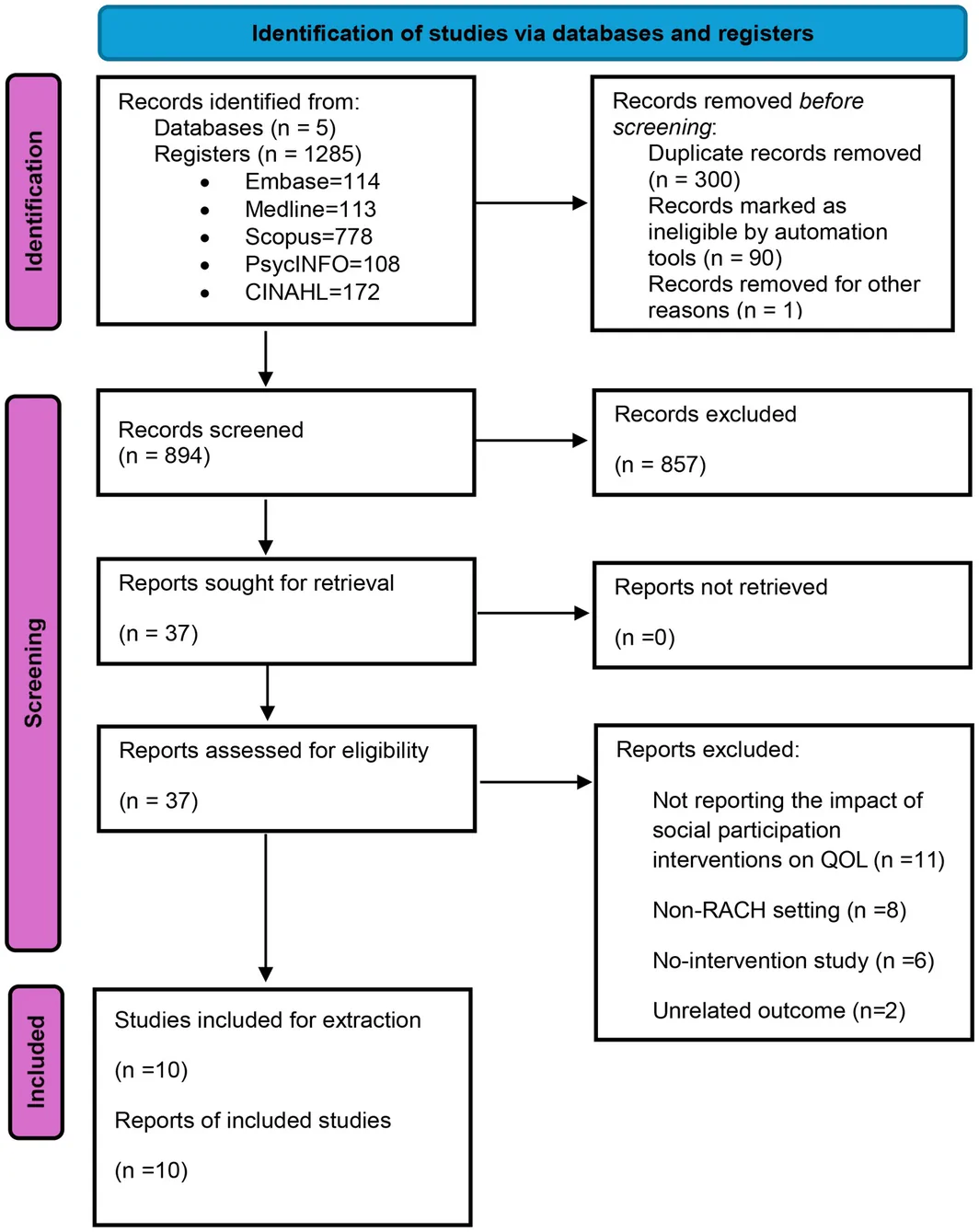
The PRISMA flowchart. [Colour figure can be viewed at wileyonlinelibrary.com]

### 3.2 Study Characteristics

Table 1 provides a summary of included studies. Of the 10 studies, six papers (60%) were conducted in Europe, followed by two (20%) in North America and two (20%) in Asia. The study designs included five randomised controlled trials (RCTs), three cluster RCTs, one quasi‐experimental, and one mixed‐method study combining quantitative and qualitative quasi‐experimental approaches. Notably, two studies did not include a control group (Fiocco et al. 2021; Wilks et al. 2019).

**TABLE 1:** Summary of included studies.

Authors	Country/Region	Number of facilities	Sample size	Participants mean age (years) and sex (% of females)	Study design	Interventions	Participants' cognitive abilities
Hsu et al. (2016)	Taiwan	1	60	Age: 81.25 ± 8 Sex: 63.3	Randomised control trial	Tai Chi exercise	Normal cognition, mild dementia
Ballard et al. (2016)	United Kingdom	16	277	Age: 85.26 ± 7.02 Sex: 73.9	Cluster randomised control trial	Person‐centred care in combination with antipsychotic review, social interaction and exercise interventions	Mild dementia, moderate dementia, severe dementia
Barthalos et al. (2016)	Hungary	1	45	Age: 77.17 ± 10.42 Sex: NS	Randomised control trial	Resistance training	Mild dementia, moderate dementia
Ballard et al. (2018)	United Kingdom	69	847	Age: 86.6 ± 0.53 Sex: 70.3	Cluster randomised control trial	WHELD program, a combination of staff training, social interaction, and guidance on use of antipsychotic medications	Mild dementia, moderate dementia, severe dementia
Husebø et al. (2019)	Norway	33	545	Age: 86.7 ± 7.5 Sex: 74	Randomised control trial	COSMOS trial, a combination of communication, systematic pain management, medication review, organisation of activities, and safety	Normal cognition, mild dementia, moderate dementia, severe dementia
Klapwijk et al. (2018)	Netherlands	12	288	Age: 83.8 ± 7.15 Sex: 71.85	Cluster randomised control trial	STA OP! trial, a multicomponent intervention consisting of six steps for assessing basic care, pain and physical needs, administering a trial of pharmacological and nonpharmacological and consulting with other disciplines, or starting a trial of prescribed psychotropic drug	Moderate dementia, severe dementia
Lai et al. (2018)	China	4	111	Age: 84.6 ± 7.24 Sex: 65.6	Randomised control trial	Horticultural therapy	Normal cognition, mild dementia
Nawrat‐Szołtysik et al. (2019)	Poland	4	91	Age: 81 Sex: 100	Randomised control trial	Modified Sinaki exercises and Nordic Walking	Did not assess
Wilks et al. (2019)	USA	1	43	Age: 86.3 ± 7.6 Sex: 74	Quasi‐experimental design	Montessori‐based activities	Severe dementia
Fiocco et al. (2021)	Canada	6	18	Age: 83.6 ± 6.17 Sex: 38.9	Mixed methods study	Virtual reality tourism exposure	Normal cognition

Abbreviation: NS, not stated.

Several studies utilised person‐centred care strategies (Wilks et al. 2019; Ballard et al. 2018, 2016; Husebø et al. 2019; Klapwijk et al. 2018), while others applied one intervention across all participants (Fiocco et al. 2021; Barthalos et al. 2016; Nawrat‐Szołtysik et al. 2019; Hsu et al. 2016; Lai et al. 2018). Four studies applied physical exercise as a type of social intervention (Ballard et al. 2016; Barthalos et al. 2016; Nawrat‐Szołtysik et al. 2019; Hsu et al. 2016). The duration of interventions and follow‐up periods ranged from 6 to 48 weeks.

In total, the 10 studies involved 2325 participants from 147 RACHs, with sample sizes ranging from 18 to 847 participants. Apart from one study (mean age: 77) (Barthalos et al. 2016), the mean age of study participants in each of the other studies was over 80 years. For all studies except one study (Fiocco et al. 2021), more than 60% of participants were female.

Cognitive function was assessed in all studies except one (Nawrat‐Szołtysik et al. 2019), using various measurement tools. Approximately 50% of the studies included participants with a wide range of cognitive abilities (Ballard et al. 2018, 2016; Husebø et al. 2019; Klapwijk et al. 2018; Barthalos et al. 2016). One study focused exclusively on individuals with severe dementia (Wilks et al. 2019), while three studies included normal cognition or mild dementia participants only (Fiocco et al. 2021; Hsu et al. 2016; Lai et al. 2018).

### 3.3 Study Interventions

The included studies investigated a diverse range of interventions aimed at enhancing QOL among RACH residents, with particular focus on social participation. Some interventions were delivered directly in group settings, ensuring mandatory social engagement simply through performing tasks (Fiocco et al. 2021; Wilks et al. 2019; Barthalos et al. 2016; Nawrat‐Szołtysik et al. 2019; Hsu et al. 2016; Lai et al. 2018). Other interventions, grounded in person‐centred care, encouraged residents to initiate and improve social engagement or provided opportunities for meaningful social interaction (Ballard et al. 2018, 2016; Husebø et al. 2019; Klapwijk et al. 2018).

The study interventions included various forms of physical exercise such as Tai Chi, resistance‐training, modified Sinaki exercises, and Nordic walking. Several studies implemented multicomponent interventions combining person‐centred care with antipsychotic medication review, social interaction, and exercise interventions. Notable programs included the WHELD (Well‐being and Health for People with Dementia) program combining staff training, social interaction, and guidance on use of antipsychotic medications, STA OP! trial (STA OP! is a Dutch abbreviation for *STA*psgewijs *O*nbegrepen gedrag en *P*ijn bij dementie de baas!) which is a stepwise multicomponent intervention and multiple assessments followed by consultation with other health professionals for both non‐pharmacological and pharmacological treatments, and the COSMOS trial (a combination of Communication, Systematic pain management, Medication review, Organisation of activities, and Safety). Other interventions included virtual reality tourism, horticultural therapy, and Montessori‐based activities (Table 1).

### 3.4 Study Outcomes

A description of the studies' QOL assessment tools, outcomes, comparator groups, outcome measurement times, and effects is presented in Table 2. Six studies reported only on QOL domains, rather than overall QOL (Ballard et al. 2016; Husebø et al. 2019; Klapwijk et al. 2018; Barthalos et al. 2016; Nawrat‐Szołtysik et al. 2019; Hsu et al. 2016). Four studies additionally assessed changes in social participation over time using dedicated tools (Fiocco et al. 2021; Wilks et al. 2019; Barthalos et al. 2016; Lai et al. 2018). Although various limitations were noted across the articles, the most common was a small sample size, mentioned in three of the studies (Fiocco et al. 2021; Wilks et al. 2019; Lai et al. 2018).

**TABLE 2:** Description of the methods employed in each included study.

	QOL assessment tool	Outcomes	Comparator groups	Outcome measurement times	Effects
Hsu et al. (2016)	WHOQOL‐BREF	Overall QOL, QOL domains: general health, physical capacity, psychological well‐being, social relationship, environment	**Intervention group** (*n* = 30): 40 min of seated Tai Chi exercise, three times a week for 26 weeks. **Control group** (*n* = 30): Usual standard care without seated Tai Chi exercise.	Baseline, 13 weeks and 26 weeks	**Effect of intervention on QOL**: **Baseline:** Significantly higher score on the general health domain in intervention group (*p* = 0.03) than control group. **At 13 weeks:** Significant positive effect on physical (*p* = 0.01) and psychological health (*p* = 0.02) domains compared to control group. **At 26 weeks:** Significant positive effect in overall QOL (*p* = 0.03) compared to control group. **Baseline to week 26:** Significant positive effect in the social relations domain (*p* = 0.01) compared to control group.
Barthalos et al. (2016)	WHOQOL‐OLD (Hungarian version)	QOL domains: sensory abilities; autonomy; past, present and future activities; social participation; death and dying; intimacy	**Intervention groups**: (i) Training group (*n* = 11): Resistance training twice a week for 45 min. (ii) Mental group (*n* = 11): Training group activities + weekly lectures/group discussions on ageing and quality of life **Control group** (*n* = 11): No physical or mental training.	Baseline and 15 weeks	**Effect of intervention on QOL:** Significant positive effect on social participation domain (**compared to baseline**): (i) Training group: (*p* = 0.004) (ii) Mental group: (*p* = 0.004). There were no significant differences between the two intervention groups.
Ballard et al. (2016)	DEMQOL‐Proxy	Overall QOL and QOL domains: cognition, negative emotions, positive emotions, daily activities, appearance	**Antipsychotic review group** (*n* = 146): Used antipsychotic medication guidelines and additional supporting educational resources to reduce antipsychotic use. **Non‐antipsychotic medication review groups** (*n* = 131): (i) Social interaction group: at least 1 h/week of social interaction (or increased social interaction by 20%). (ii) Exercise group: physical activity, at least 1 h/week (or 20% more than at baseline).	Baseline and 36 weeks.	**Effect of non‐antipsychotic intervention on QOL:** (i) Social interaction group: Significant positive effect on overall QOL (*p* = 0.042), cognition domain (*p* = 0.025) and appearance domain (*p* = 0.009) compared with other groups. (ii) Exercise group: No effect on overall QOL (*p* = 0.334), but significant positive effect on positive emotion domain (*p* = 0.001) compared with other groups. **Effect of antipsychotic intervention on QOL:** Significant negative effect on negative emotion (*p* = 0.02) and appearance (*p* = 0.04) domains compared with other groups. **Effect of both antipsychotic review and social interaction on QOL:** No impact (*p* = 0.62) compared with those receiving neither of these interventions.
Ballard et al. (2018)	DEMQOL‐Proxy	Overall QOL	**Intervention (WHELD) group** (*n* = 257): WHELD programme includes combined staff training, social interaction, and guidance on use of antipsychotic medications. **Control (TAU) group** (*n* = 296): Treatment as usual.	Baseline and 36 weeks.	**Effect of intervention on QOL:** Significant positive effect (*p* = 0.0042) compared to control group.
Husebø et al. (2019)	QUALIDEM QUALID EQ‐VAS	Overall QOL	**Intervention group** (*n* = 297): Health care staff received 4 months of training in the COSMOS program for implementing the multicomponent trial. They were then required to provide a minimum of 90 min of personalised engagement per week.; for residents who had already received 90 min of personalised engagement, the goal of the intervention was to increase the activities by 25% beyond this baseline. **Control group**(*n* = 248): Usual care.	Baseline, 16 weeks and 36 weeks.	**Effect of intervention on QOL**: **Baseline to 16 weeks:** Significant negative effect for both QUALID (*p* = 0.04) and QUALIDEM (*p* = 0.002) compared to control group. **16–36 weeks:** Significant positive effect for both EQ‐VAS (*p* = 0.003) and QUALIDEM (*p* = 0.012). **Baseline to 36 weeks:** No significant effect in QUALIDEM, QUALID And EQ‐VAS compared to control group (*p* > 0.05).
Klapwijk et al. (2018)	QUALIDEM	QOL domains: care relationship, positive affect, negative affect, restless tense behaviour, social relations, and social isolation	**Intervention group** (*n* = 148): Staff training on challenging behaviour in dementia and pain management + stepwise multicomponent intervention (STA OP!) including: (1) assessments for: basic care needs, pain and physical needs, affective needs; (2) nonpharmacological comfort treatment; (3) analgesic agents; (4) psychotropic drugs. Several structured interactions occurred between staff and residents to develop personalised activity plans as a non‐pharmacological approach. **Control group** (*n* = 140): Staff training on challenging behaviour in dementia and pain management.	Baseline, 12 weeks and 24 weeks.	**Effect of intervention on QOL:** **Baseline to 12 weeks:** Significant positive effect in restless tense behaviour domain (β: 0.95) * compared to control group. **12–24 weeks:** Significant negative effect in restless tense behaviour domain (β: −0.98) * and significant positive effect in social isolation domain (β: 0.64) * compared to control group.
Lai et al. (2018)	PWBI	Wellbeing	**Intervention group** (*n* = 56): Horticultural therapy 60 min/week, gardening for 8 weeks. **Control group** (*n* = 55): Activities that did not include the use of plants or plant‐related materials, for example, talking about the news, card games.	Baseline, immediately post‐intervention, and 12 weeks post‐intervention.	**Effect of intervention on QOL:** No significant between groups.
Nawrat‐Szołtysik et al. (2019)	QUALEFFO‐41 questionnaire	QOL domains: pain, activities of daily living, jobs around the house, mobility, leisure and social activities, general health perception, and mental function	**Intervention groups**: Group 2 (*n* = 23): Modified Sinaki exercises in a group setting, twice a week + drug therapy Group 3 (*n* = 21): Nordic walking, twice a week + drug therapy Group 4 (*n* = 23): Sinaki exercises in group setting + Nordic walking +drug therapy. **Control group** (Group 1) (*n* = 24): only drug therapy	Baseline and 48 weeks.	**Effect of intervention on QOL:** Significant positive effects were experienced by all intervention groups (*p* = 0.02) Thus, the intervention groups overall experienced improvements and each of the individual interventions had a significant positive effect on QOL. (*p* = 0.00). QOL was significantly better in groups **2** (*p* = 0.01) and **4** (*p* < 0.01) compared to other groups. Groups **2 and 4**: Significant positive effect (*p* ≤ 0.05) in the leisure and social activities domain compared to other groups. Group **1 and 3**: Significant negative effect (*p* ≤ 0.05) in the leisure and social activities domain compared to other groups. The effect of particular therapies (Sinaki exercise) was statistically significant (*p* = 0.01).
Wilks et al. (2019)	QoL‐AD	Overall QOL	**Intervention group** (*n* = 43): Montessori‐based activities, for example, intellectually stimulating exercises, sensory puzzles and games, group readings, and engagement in household activities. There was no Control group.	Baseline, 12 weeks and 24 weeks.	**Effect of intervention on QOL**: **Baseline to 12 weeks:** Significant positive effect (*p* < 0.05). **12–24 weeks:** Significant negative effect (*p* < 0.01). **Baseline to 24 weeks:** Not significant (*p* = 0.66).
Fiocco et al. (2021)	OPQOL‐35	Overall QOL	**Intervention group** (*n* = 18): 6–10 min Virtual reality tourism sessions, three times/week for 6 weeks. Each session used three headsets for three participants, with two research assistants present. There was no Control group.	Baseline and 6 weeks.	**Effect of intervention on QOL**: Significant positive effect (*p* < 0.001) compared with baseline.

Abbreviations: n, number; h, hour; min, minute; QOL, quality of life; TAU, treatment as usual; WHELD, Well‐being and Health for People with Dementia.

* Positive β indicates a significant positive effect, whereas negative β indicates a significant negative effect.

The choice of QOL assessment tools was based on the participants' cognitive and functional characteristics. For example, the DEMQOL‐proxy was utilised for participants who could not complete the assessment themselves. Notably, one study also assessed the impact of the intervention on care staff and primary caregivers (Husebø et al. 2019). All QOL assessment tools used in the included studies are summarised in Table 3.

**TABLE 3:** Summary of QOL tools used in included studies.

Tool name	Description
DEMQOL‐Proxy	Dementia Quality of Life Questionnaire (proxy version): 31 items assessing QOL in dementia patients
EQ‐VAS	EuroQOL Visual Analogue Scale: 1 item (scale 0–100) capturing self‐rated health status
OPQOL‐35	Older People's Quality of Life Questionnaire: 35 items evaluating subjective QOL
PWBI	Personal Well‐being Index: 9 items assessing satisfaction across various life domains
QOL‐AD	Quality of Life in Alzheimer's Disease: 13 items measuring QOL from the perspective of individuals with Alzheimer's or their caregivers
QUALEFFO‐41	Quality of Life Questionnaire of the European Foundation for Osteoporosis: 41 items
QUALID	Quality of Life in Late‐Stage Dementia: 11 items designed for individuals with severe/late‐stage dementia
QUALIDEM	Quality of Life in Dementia: 37 items for people with dementia in long‐term care settings
WHOQOL‐BREF	World Health Organisation Quality of Life – BREF (abbreviated version): 26 items across 6 domains
WHOQOL‐OLD (Hungarian version)	World Health Organisation Quality of Life – Older Adults Module (Hungarian version): 24 items

### 3.5 Impact of Social Participation Interventions on RACH Residents' Quality of Life

All included studies reported on the impact of their interventions on overall QOL or specific QOL domains. Significant improvements in QOL were observed following various interventions, including a resistance training‐based physical activity (Barthalos et al. 2016), a program of modified Sinaki exercises and Nordic walking (Nawrat‐Szołtysik et al. 2019), Tai Chi exercise (Hsu et al. 2016), person‐centred care and psychosocial intervention (antipsychotic medication review and WHELD) (Ballard et al. 2018, 2016), and Virtual reality tourism exposure (Fiocco et al. 2021). For example, Ballard et al. reported wide‐ranging QOL improvements from the combined implementation of person‐centred care, antipsychotic review, social interaction, and exercise interventions. This study included several intervention groups. While no overall QOL improvements were noted in the exercise group of the non‐antipsychotic review intervention, a significantly positive change was reported in the social interaction group. The impact of antipsychotic review was negative on QOL, but through combining antipsychotic review with social interaction intervention, this negative influence on QOL was not observed (Ballard et al. 2016).

Several studies reported fluctuating or non‐significant impacts of interventions on QOL over time, highlighting the complexity and variability in outcomes among RACH residents. For example, Klapwijk et al. (2018) observed QOL enhancement in restlessness/tension during the first 12 weeks of the STA OP! intervention, which declined during the subsequent 12 weeks. However, the social isolation domain of QOL improved between the 12 to 24 week follow‐up. Similarly, Husebø et al. (2019) found a decrease in QOL in the first 16 weeks, which then reversed by the following 20 week follow‐up. Wilks et al. (2019) demonstrated QOL enhancement during the first 12 weeks, followed by deterioration in the next 12 weeks. However, the impact of the interventions (COSMOS trial and Montessori‐based activities) from baseline to final follow‐up was not statistically significant (Wilks et al. 2019; Husebø et al. 2019). Likewise, Lai et al. (2018) did not report any significant change in the participants' QOL with a Horticultural intervention.

Several studies demonstrated a positive association between increased social participation and improvements in QOL, either as a direct outcome of the intervention or as a contributing factor to observed QOL changes. For instance, Fiocco et al. (2021) found that virtual reality tourism not only showed positive changes in social participation and QOL, but also a strong correlation between increasing social participation and QOL. Barthalos et al. revealed a positive association between social participation and QOL. This was observed in both the mental and training groups that had exercise combined with group discussion or lectures, and supervised exercise alone, respectively, when compared to baseline (Barthalos et al. 2016), while Nawrat‐Szołtysik et al. (2019) reported higher QOL among participants who performed Sinaki exercises together versus those doing Nordic walking individually. Ballard et al. (2016) also demonstrated that combining social interaction with person‐centred care was an effective approach to improving QOL and a progressive step in addressing RACH residents' challenges, particularly when combined with antipsychotic reviews.

Further reinforcing the relationship between social participation and QOL, three studies suggested that improvements in QOL may be mediated by increased social participation. The findings of Ballard et al. (2018) based on the WHELD intervention concluded that enhanced social participation may contribute to the observed improvements in QOL, particularly among those with moderately severe dementia. Similarly, Klapwijk et al. (2018) observed a reduction in the social isolation aspect of QOL, implying that increased social participation could result in QOL enhancement. Finally, Hsu et al. (2016) revealed that Tai Chi exercise had a significantly improved perceived QOL among residents in RACH, which could be primarily associated with a significant increase in social participation. By contrast, three studies did not report any significant association between social participation and QOL outcomes (Wilks et al. 2019; Husebø et al. 2019; Lai et al. 2018).

### 3.6 Quality Appraisal of Studies

The methodological quality of the included studies, as assessed using the MMAT, is summarised in Table 4, with most studies met the majority of methodological criteria, although some limitations were identified. Specifically, three studies met all applicable MMAT criteria (Fiocco et al. 2021; Nawrat‐Szołtysik et al. 2019; Lai et al. 2018), indicating no identified methodological limitations based on the tool. The most common methodological limitations across studies were incomplete outcome data (*n* = 4) due to participant attrition (e.g., death) (Ballard et al. 2018, 2016; Husebø et al. 2019; Klapwijk et al. 2018), lack of blinding (*n* = 2) (Barthalos et al. 2016; Hsu et al. 2016), and lack of baseline participant comparability (*n* = 1) (Klapwijk et al. 2018).

**TABLE 4:** Quality appraisal of included studies.

	Q1	Q2	Q3	Q4	Q5	Category of study design
Hsu et al. (2016)	Yes	Yes	Yes	No	Yes	Quantitative randomised controlled trials
Barthalos et al. (2016)	Yes	Yes	Yes	Can not tell	No	Quantitative randomised controlled trials
Ballard et al. (2016)	Yes	Yes	No	Yes	Yes	Quantitative randomised controlled trials
Ballard et al. (2018)	Yes	Yes	No	Yes	Yes	Quantitative randomised controlled trials
Husebø et al. (2019)	Yes	Can not tell	No	Yes	Yes	Quantitative randomised controlled trials
Klapwijk et al. (2018)	Yes	No	No	Yes	Yes	Quantitative randomised controlled trials
Lai et al. (2018)	Yes	Yes	Yes	Yes	Yes	Quantitative randomised controlled trials
Nawrat‐Szołtysik et al. (2019)	Yes	Yes	Yes	Yes	Yes	Quantitative randomised controlled trials
Wilks et al. (2019)	Yes	Yes	Yes	Yes	No	Quantitative non‐randomised
Fiocco et al. (2021)	Yes	Yes	Yes	Yes	Yes	Mixed methods

Abbreviation: Q, question.

## 4 Discussion

Findings from this review suggest that various social participation interventions can significantly enhance QOL among RACH residents. The most common social participation interventions were non‐exercise and indoor activities, mainly due to participants' physical limitations and safety concerns. Despite variations in intervention types, benefits to QOL were observed across physical, psychosocial, and creative engagement activities. Among several limitations, the small sample size influenced the effect measures and reduced the generalisability of the results.

While many interventions demonstrated positive effects, some studies observed no or adverse impacts on their outcomes. For example, while a recent meta‐analysis concluded that horticultural therapy is an effective intervention to enhance QOL in older adults (Lin et al. 2022), Lai et al. did not report QOL and social participation enhancement after this intervention but they found a significant increase in participants' happiness. A small sample size (*n* = 56 under 80% of calculated sample size) and the short duration of the intervention (a total of eight hours, delivered as one hour per week) may have contributed to these results and reduced the effect size (Lai et al. 2018). Similarly, Nawrat‐Szołtysik et al. (2019) reported that while Nordic walking improved QOL, it had a negative impact on social participation, likely due to the lack of a social participation component, unlike the modified Sinaki exercise, which was a group‐based program.

Several methodological challenges further affected study outcomes. In particular, studies with small samples, absence of control groups, or inclusion of participants in advanced stages of dementia may have been particularly vulnerable to confounding factors (Wilks et al. 2019). For example, the findings of Wilks et al. on the Montessori intervention for participants with severe dementia showed a decline in social participation since the programme focused on individual tasks and did not allow enough time for communication or social engagement. Although QOL fluctuated at various measurement times during the study period, the overall positive change was small and not statistically significant. A recent systematic review indicated that the rapid decline in physiological and cognitive abilities of participants with late‐stage dementia may have contributed to the reduction of effectiveness of the Montessori‐based programmes (Yan et al. 2023).

Several studies highlighted the broader effects of their interventions beyond direct changes in social participation. For example, Ballard et al. (2016) reported that QOL improved in the social interaction group and adding a social interaction intervention to an antipsychotic medication review had a protective effect and prevented QOL decline in their participants. In addition, they observed a statistically significant benefit in positive care interactions (Ballard et al. 2018) which shows the potential value of social participation intervention on enhancing QOL. Klipwijik et al. reported an improvement in the social isolation domain. They suggested that reducing social isolation could lead to increased social interaction and, in turn, enhance QOL. However, they also noted that their results may be subject to potential bias due to the lack of blinding between the control and intervention groups (Klapwijk et al. 2018).

The variation between study results across different measurement times highlights the importance of follow‐up duration. For instance, Husebø et al. (2019) and Wilks et al. (2019) reported fluctuations in their outcomes over time, including short‐term improvements or declines, although their final results did not show significant changes in the long term. To improve the accuracy of judging an intervention's effectiveness, it is important to extend the follow‐up evaluation period. Additionally, measuring outcomes throughout the study helps determine at which time points the intervention may be most effective.

Heterogeneity in the cognitive abilities of participants can affect the effect size of the intervention. For example, Ballard et al. (2018) found the same statistically significant benefits of the intervention on QOL only among participants with moderately severe dementia. Similarly, Wilks et al. (2019) explained their findings by noting that their study included only participants with severe dementia. In contrast, Lai et al. (2018) studied individuals with normal cognition or only mild dementia, which may explain why their results were not statistically significant.

Evaluating QOL in older adults, particularly those with dementia, remains challenging, despite the use of validated methods (Ballard et al. 2018) since the validity of measurement tools varies; interpreting the results can be difficult. For example, the use of the QUALIDEM total score is subject to debate due to concerns about its lack of validation (Klapwijk et al. 2018). Additionally, using proxy‐rated QOL measures may introduce observation bias (Ballard et al. 2016), especially when the outcome assessor is not blinded to the treatment group. This can reduce the reliability of the results compared to self‐reported QOL measures.

### 4.1 Strengths and Limitations

To the best of our knowledge, this systematic review is the first investigation to review current literature on the impact of social participation interventions on QOL among older adults residing in RACHs. Searching five major databases, consulting a clinical librarian, and including only peer‐reviewed journals strengthens this study. Additionally, the use of the MMAT allows for a consistent assessment of the methodological quality of all included studies. However, including a wide range of study designs strengthens the validity and reliability of the findings; it also limits the generalisability of the results across specific intervention types and participants' characteristics.

The study is also associated with several limitations. It is difficult to make direct comparisons between study findings due to heterogeneity across included studies. The authors reported the effectiveness of interventions in several ways across the included studies. The reporting of intervention effectiveness varied considerably across the included studies. Significant variability in QOL assessment tools further complicated interpretation. Some studies reported the overall QOL score through a wide range of QOL assessment tools, while others relied on several domains within QOL assessment tools. In some cases, researchers interpreted their findings as indicative of effectiveness, even without having statistically significant results. The inconsistency in reporting methods, measurement tools and interpretations across the studies limits the ability to draw strong, comparable conclusions.

Many relevant articles identified through citation searching were qualitative studies and were excluded due to the lack of quantitative results; therefore, we missed the important perspective and findings of these studies. Blinding of participants was also difficult, as both intervention and control groups resided in the same RACH. Furthermore, gender balance was an often‐overlooked factor that can reduce the generalisability of study findings. We could not conduct a meta‐analysis or determine the most effective intervention due to variations in sample sizes, participant characteristics, cultural contexts, follow‐up periods, and assessment tools. Furthermore, limiting the inclusion criteria to English‐language publications potentially introduced selection bias and limited the generalisability of the results.

### 4.2 Research Gap and Future Challenges

This study shows that social activities can influence several QOL domains. Therefore, it is recommended that future studies report not only overall QOL but also results across specific QOL domains. This would allow more targeted interventional approaches; for example, if a provider wanted to specifically target the independence domain of QOL, they could identify those interventions that showed a positive impact on this specific domain and trial the implementation of that intervention.

Gender differences in QOL should be considered when selecting study participants, as the impact of social participation on QOL may vary between males and females. A study by Lee et al. (2020) showed that male older adults perceived social relationships as more important than family relationships, unlike female older adults. Their findings showed that social cohesion was significantly associated with higher QOL among males compared to females. Since most studies included in this current study had more than 60% female participants, representing the dominance of the female population in RACHs, future studies should aim for a more balanced gender distribution to improve the reliability and generalisability of findings.

Social participation interventions should be meaningful and motivate RACH residents to participate, such as Tai Chi (Chen et al. 2002). Culturally significant interventions not only may increase the effectiveness of activities by making them more meaningful for both older adults and staff, but they can also be incorporated into participants' daily routines, which enhances the feasibility of intervention in RACH settings. Therefore, future research should prioritise culturally significant interventions embedded into routine aged care practices. Additionally, larger sample sizes and longer‐term follow‐up will help to generate more robust evidence to support the impact of social participation interventions on QOL improvements in aged care settings.

## 5 Conclusions

This systematic review revealed that improvements in social participation and QOL may be associated with various social participation interventions such as virtual reality tourism, Tai Chi exercise, resistance training, Sinaki exercises, and programs involving pain management, communication, organisation of activities and medication review. Multiple studies showed a positive correlation between increased social participation and enhanced QOL among RACH residents. However, due to substantial heterogeneity of interventions and the absence of a meta‐analysis, these findings should be interpreted with caution. As some interventions were found to be effective for both social participation and QOL, they may be considered for implementation in RACH by considering residents' characteristics and preferences to maximise their benefits.

## Funding

This study was supported by the International Macquarie University Research Excellence Scholarship (‘iMQRES’ scholarship allocation number 20246959).

## Conflicts of Interest

The authors declare no conflicts of interest.

## Supporting information


**Table S1:** The search strategy.
**Table S2:** The full search strategy for MEDLINE (Ovid).

## Data Availability

No new data were generated or analysed in this study. All data are derived from previously published studies and are included in the article and its Supporting Materials.
