# Supplementary material for: The Impact of Social Participation Interventions on Quality of Life of Older Adults in Residential Aged Care Facilities: A Systematic Review
Source: J Clin Nurs. 2026 Aug 4;35(12):5355–70. doi: 10.1111/jocn.70483 (PMC13744227; doi:10.1111/jocn.70483)
Supplement: Supplementary file 1 [file JOCN-35-5355-s001.docx]

Supplementary Table 1: The search strategy

| **CONCEPT 1- POPULATION/SETTING** | **CONCEPT 2- INTERVENTIONS** | **CONCEPT 3-OUTCOME** |
| --- | --- | --- |
| Aged care facilit* | Social engagement* | Qualit* of life |
| Residential aged care/facilit* | Social activit* | Wellbeing |
| Nursing home* | Social involvement* | Quality adjusted life year* |
| Long term care | Social participation* | Health related quality of life |
| Care facilit* | Social interaction* | Life qualit* |
| Residential care | Community engagement* | Well being |
| Home* for the aged | Community activit* | Well-being |
|  | Community involvement* |  |
|  | Community participation* |  |
|  | Community interaction* |  |

Supplementary Table 2: The full search strategy for MEDLINE (Ovid)

| Step | Search query |
| --- | --- |
| 1. | "quality of life"/ or well-being/ or wellbeing/ or "well being"/ |
| 2. | ("qualit* of life" or "quality adjusted life year*" or "well being" or "health related quality of life" or "life qualit*" or "well-being" or "wellbeing").ti,ab. |
| 3. | Homes for the Aged/ |
| 4. | nursing homes/ |
| 5. | ("residential aged care/facilit*" or "nursing home*" or "aged care facilit*" or "care facilit*" or "home* for the aged" or "residential care" or "long term care").ti,ab. |
| 6. | social participation/ |
| 7. | ((social or community) adj2 (participation or engagement* or interaction* or activit* or involvement*)).ti,ab. |
| 8. | 6 or 7 |
| 9. | 1 or 2 |
| 10. | 3 or 4 or 5 |
| 11. | 8 and 9 and 10 |
| 12. | limit 11 to (english language and yr="2014 - 2024" and "all aged (65 and over)" and english and elderly - focussed) |
